# The Fish Theory of Leprosy

**Published:** 1902-01-11

**Authors:** 


### THE FISH THEORY OF LEPROSY.

Writing on the subject of the fish hypothesis as to the origin of leprosy, Mr. Jonathan Hutchinson [1] says that those who support this theory by no means suspect all kinds of fish, and do not hold that a large consumption of any one kind is essential to the result. The kind of fish food under chief suspicion is dried or imperfectly salted fish. Fish in one of these conditions is often imported inland. It is light, indestructible, and easy of carriage, and it is in great request as a condiment wherever the supply of fresh meat is deficient. Especially is it valued wherever rice is largely eaten, and it is often used in conditions of decomposition which would render it very repulsive to European palates. It is suspected then that fish food of this kind, uncooked, may either carry the bacillus itself, or be the stimulant which excites it to action. Some would even go as far as to suggest that it may perhaps transmute the tubercle bacillus into that of leprosy. In regard to the facts brought up in favour of the fish theory we need not now speak; what is more important is how Mr. Hutchinson gets over the objections which have been raised to it. He gives three chief objections as being those of most importance, and answers them as follows:—

(1) Its non-occurrence at the present day amongst many communities which yet consume fish freely. In reply to this it may be admitted that if sporadic cases did occur more frequently in non-leprous districts, and if these could be traced to salt fish eating the hypothesis would be strengthened; but a fair answer to the objection is that the fish which is now consumed in civilised communities is either fresh or well cured, and that it is usually well cooked.

(2) Its occurrence in many inland communities who do not consume any large quantity of fish. This objection has usually been urged in forgetfulness of the fact that it is not the consumption of large quantities which is under suspicion, but of small quantities of a bad kind.

(3) The fact that some individual lepers will deny that they have ever tasted fish. This kind of evidence comes almost solely from India, and it is given chiefly by those who by religious creed are forbidden to eat it. In many instances it may have been that the inquiry was not make definite as regards fish condiments and dried fish, and it may have been in some that the reply was not a truthful one. These are the replies given by Mr. Hutchinson to the objection raised.

We cannot, however, shut out the fact that the acceptability of the fish theory depends largely upon the fact that there is no other workable hypothesis in the field. "No one," says Mr. Hutchinson, "can assign any other plausible conjecture as to why a disease which is identical in all races should prevail all over the world, and should have done so from the earliest periods of history, being apparently indigenous in the most distant places and under the most dissimilar conditions. No other article of food is of universal consumption." We confess that we should like to see the microbe in the fish.

[1] Polyclinic, December, 1901.

